# Benchmarking Drug Library Compliance and Alert Rates in Smart Infusion Pumps: A Multicenter Retrospective Study

**DOI:** 10.7759/cureus.116103

**Published:** 2026-09-11

**Authors:** Ivens Souza, Mona Mourad, Samantha J Burton, Jaculin Cole, Caio Plopper, Sofia R Fernandez, Aftab Alam

**Affiliations:** 1 Medical Affairs, Baxter Healthcare Corporation, Deerfield, USA; 2 Medical Affairs, Baxter Healthcare Corporation, Mississauga, CAN

**Keywords:** alert fatigue, benchmarking, clinical decision support, continuous quality improvement, dose error reduction software, drug library compliance, medication safety, smart infusion pumps

## Abstract

Introduction: Smart infusion pumps with dose error-reduction software (DERS) generate alerts to prevent infusion programming errors. However, high alert volumes may contribute to alert fatigue and impact workflow efficiency. Benchmarking continuous quality improvement (CQI) data across institutions may help identify opportunities to optimize drug library (DL) performance. This study evaluated the relationship between DL compliance and alert-related metrics using a benchmarking approach.

Methods: A retrospective multicenter analysis of smart infusion pump data was conducted across 90 institutions (99 data pulls), including 13,749,759 infusions. Key metrics included DL compliance, alert rate, override rate, and alerts resulting in reprogramming (“good catches”). Facilities with DL compliance <95% and ≥95% were compared, and Spearman correlation analyses were performed to assess associations between DL compliance and alert-related outcomes.

Results: The overall DL compliance rate was 95.9%. The mean alert rate was 33.3% (±12.20), and the overall override rate was 96.4%. On average, one good catch occurred for every 34.5 DERS infusions. Benchmarking across institutions showed no statistically significant differences in alert rates, override rates, or good catch rates between facilities with DL compliance ≥95% and those with <95%. Correlation analyses demonstrated only weak associations between DL compliance and alert rate, override rate, and good catch metrics.

Conclusions: Benchmarking CQI data across institutions demonstrates that high DL compliance alone does not necessarily indicate optimized smart infusion pump performance. Incorporating alert and override metrics into CQI analyses may help reduce non-actionable alert burden and support safer, more efficient infusion workflows.

## Introduction

Smart infusion pumps equipped with dose error-reduction software (DERS) are widely used to enhance medication safety. In 2018, 89.5% of hospitals in the United States reported using smart pump technology [1]. These systems integrate clinical decision support with a customized drug library (DL) containing standardized drug concentrations, dosing parameters, and safety limits. DERS generates alerts when infusion parameters fall outside predefined limits, with the goal of preventing infusion programming errors and supporting safer medication administration [2-4].

DERS includes both hard and soft limit alerts. Hard limits require clinicians to reprogram the infusion or discontinue DERS use, preventing administration outside established safety parameters. In contrast, soft limit alerts and single-step rate change (SSRC) alerts provide warnings that can be overridden, allowing the infusion to continue after user confirmation [5].

Although these smart infusion pump features have been associated with reductions in medication errors and adverse events, excessive alerts may contribute to alert fatigue. Frequent exposure to non-actionable alerts can lead to desensitization, resulting in high override rates and reduced effectiveness of safety systems [6-8]. Failure to appropriately respond to clinically meaningful alerts may increase the risk of patient harm [9,10].

DLs require ongoing maintenance and optimization to balance safety and usability [2]. DL compliance, defined as the proportion of infusions programmed using DERS rather than basic mode, is commonly used as a key performance metric in continuous quality improvement (CQI) programs [5]. While low compliance may reduce the effectiveness of smart pump safety features [2], high compliance alone does not necessarily indicate an optimized DL or reduced alert burden. Achieving optimal performance requires balancing safety limits with clinical workflow considerations [4].

Smart infusion pump data provide valuable opportunities for CQI analysis, including evaluation of alert types, frequencies, override behavior, and reprogramming events [5,6]. Benchmarking these metrics across institutions may help identify opportunities to optimize DL configuration and reduce non-actionable alerts.

The objectives of this study were twofold: (1) to benchmark smart infusion pump CQI metrics across multiple institutions, including DL compliance, alert rates, override rates, and alerts resulting in reprogramming ("good catches"); and (2) to evaluate associations between DL compliance and these alert-related metrics.

## Materials and methods

Study design and data source

Institutions included in this retrospective multicenter study were enrolled in a smart infusion pump performance analytics program where they contributed de-identified aggregate CQI data generated from system reports specific to one infusion pump platform. Data extracts were generated using standardized reporting functionality within the infusion pump analytics platform. Institutions contributed data covering variable observation periods between January 1, 2014 and September 30, 2020. Only datasets containing at least 10,000 complete infusion records were included in the analysis.

The unit of analysis was the individual data extract. A total of 99 data extracts from 90 institutions, comprising 13,749,759 infusion records, were included. Some institutions contributed more than one extract corresponding to different observation periods. Each extract was analyzed as a separate benchmarking observation. As some institutions contributed more than one data extract, observations may not have been fully independent. Analyses were performed at the data-extract level, and potential clustering of repeated observations within institutions was not explicitly modeled.

Because the dataset contained only de-identified and aggregated quality improvement data, this study did not meet the criteria for human subjects research and institutional review board approval was not required.

Measures and definitions

The following definitions were used in the analysis: DL compliance, proportion of infusions programmed using DERS relative to total infusions; Basic mode infusions, infusions programmed manually outside the DL; Hard limit alerts, non-overridable alerts requiring reprogramming or discontinuation of the infusion when predefined limits are exceeded; Soft limit alerts, alerts indicating infusion parameters outside recommended limits that can be overridden by the clinician; SSRC alerts, alerts triggered when a programmed rate or titration exceeds a predefined percentage change from the current rate, designed to prevent large unintended changes.

Outcome measures included the following: Alert rate, total number of alerts divided by the number of DERS-enabled infusions calculated from aggregate counts captured by the infusion pump system. Override rate, proportion of soft limit and SSRC alerts where system records indicated the alert was dismissed and the infusion proceeded without reprogramming. Rate of alerts resulting in reprogramming (also defined as “good catches” [5]), proportion of alerts where system records indicated modification of the infusion parameters following an alert (including hard limit events and non-overridden soft limit and SSRC alerts). Good catches per DERS infusion, number of alerts resulting in reprogramming divided by total DERS infusions.

Statistical analysis

Descriptive statistics were used to summarize DL compliance, alert rates, override rates, and good catch metrics. Data were compared between institutions with DL compliance <95% and ≥95%, consistent with the Institute for Safe Medication Practices (ISMP) benchmarking target for smart infusion pump optimization [5]. The 95% threshold was selected a priori to facilitate comparison between institutions meeting versus not meeting the benchmark and was not intended to represent a clinical threshold.

Because several study variables demonstrated non-normal distributions and the objective was to evaluate institutional benchmarking metrics, nonparametric methods were selected. Comparisons between DL compliance groups (<95% and ≥95%) were performed using Wilcoxon two-sample tests. Associations between DL compliance and alert-related outcomes, including alert rates, override rates, and good catch metrics, were assessed using Spearman rank-order correlations. Scatter plots were used to visualize relationships between selected variables, including alert rates and override rates.

All statistical analyses were performed using SAS version 9.4 (SAS Institute Inc., Cary, NC, USA). Results were considered statistically significant at a predefined significance level of α = 0.05 (p < 0.05).

## Results

Dataset characteristics and DL compliance

A total of 13,749,759 infusion records were analyzed across 90 institutions (99 data extracts), with a mean data collection period of 370 days. The median number of infusions per day was 223 (range: 113-403). Of these, 13,207,708 infusions were programmed using DERS, resulting in a weighted overall DL compliance rate across all infusions of 96.1% (13,207,708/13,749,759). The non-weighted mean of individual institution-level DL compliance rates was 95.9%. A total of 542,051 infusions were programmed in basic mode, corresponding to a median of six infusions per day (range: three to 12). Among all data extracts, 78% (77/99) demonstrated DL compliance ≥95% (Table 1).

**Table 1:** Facility characteristics. ^a^ N corresponds to the number of data pulls. An institution could have had multiple data pulls in case they accounted for different data periods in the database. There was a total of 99 data pulls from 90 different institutions. ^b^ Non-weighted average of individual hospital DL compliance rates. Abbreviations: DL, drug library; DERS, dose error-reduction software; Min, minimum; Max, maximum

Parameter	Measure	All (N=99) ^a^	DL Compliance ≥95% (N=77)	DL Compliance <95% (N=22)
Total Infusions	Count	13,749,759	11,325,144	2,424,615
Total DERS Infusions		13,207,708	11,012,267	2,195,441
Total Infusions in Basic Mode		542,051	312,877	229,174
DL Compliance Rate (%)	Mean ^b^	95.9	97.4	90.4
	Min	73.2	95.2	73.2
	Max	99.3	99.3	94.9

Alert rates

The overall alert rate was 33.3% (±12.20), including 2.5% hard limit alerts, 10.6% soft limit alerts, and 20.3% SSRC alerts. Comparative analysis showed no statistically significant differences in alert rates (including hard, soft, and SSRC alerts) between institutions with DL compliance ≥95% and those with DL compliance <95% (Table 2).

**Table 2:** Alert rates per DERS infusion. ^a ^Grouped by DL compliance (≥95% vs. <95%); ^b ^Test statistics are reported as z-values. A two-sided p-value <0.05 was considered statistically significant. ^c^ Performed using the Wilcoxon two-sample test. Abbreviations: DERS, dose error-reduction software; SD, standard deviation; SSRC, single-step rate change

Parameter	Group ^a^	Mean (%)	SD	z-value^ b^	p-value^ c^
All Alerts	All	33.3	12.20	1.73	0.084
	≥95%	32.3	11.94		
	<95%	36.8	12.74		
Hard Limit Alerts	All	2.5	1.25	-0.11	0.916
	≥95%	2.4	1.03		
	<95%	2.6	1.86		
Soft Limit Alerts	All	10.6	7.06	0.678	0.498
	≥95%	10.5	7.40		
	<95%	10.9	5.83		
SSRC Alerts	All	20.3	8.18	1.72	0.085
	≥95%	19.4	7.41		
	<95%	23.3	10.04		

Alert override rates were high across all alert types, with an overall override rate of 96.4%, including 92.9% for soft limit alerts and 97.8% for SSRC alerts. No statistically significant differences in override rates or rates of alerts resulting in reprogramming (“good catches”) were observed between institutions with DL compliance ≥95% and those with DL compliance <95% (Table 3). 

**Table 3:** Alert overrides and alerts resulting in reprogramming. ^a^ Grouped by DL compliance (≥95% vs. <95%); ^b^ n = number of override events, N = total number of alerts in the corresponding category; ^c^ Test statistics are reported as z-values. A two-sided p-value <0.05 was considered statistically significant. ^d^ Performed using the Wilcoxon two-sample test. Abbreviations: DL, drug library; SD, standard deviation; SSRC, single-step rate change

Parameter	Group^ a^	Mean (%)	SD	n/N^ b^	z-value ^c^	p-value ^d^
Overall Override Rate	All	96.4	1.69	3,695,463/3,831,714	1.40	0.161
	≥95%	96.3	1.74	3,139,290/3,253,797		
	<95%	96.9	1.41	556,173/577,917		
Soft Limit Override Rate	All	92.9	4.46	1,176,327/1,256,976	1.22	0.221
	≥95%	92.5	4.71	992,214/1,059,957		
	<95%	94.1	3.22	184,113/197,028		
SSRC Override Rate	All	97.8	0.90	2,519,136/2,574,738	1.11	0.268
	≥95%	97.7	0.93	2,147,076/2,193,840		
	<95%	98.0	0.78	372,051/380,889		
Rate of Alerts Resulting in Reprogramming (Good Catches)	All	11.7	6.73	N/A	-0.787	0.431
	≥95%	12.0	6.85			
	<95%	10.6	6.30			

Alerts resulting in reprogramming (“good catches”)

Among DERS-enabled infusions, 425,898 (3.0%) were associated with reprogramming events (“good catches”). The overall rate of alerts resulting in reprogramming was 11.7% (±6.72) (Table 3).

The mean number of good catches was 3.3 (±1.28) per 100 DERS infusions, corresponding to approximately one good catch for every 34.5 infusions (Table 4). Among these events, 68.0% (289,650/425,898) were associated with hard limit alerts, 18.9% (80,647/425,898) with soft limit alerts, and 13.1% (55,601/425,898) with SSRC alerts.

**Table 4:** Alerts resulting in reprogramming (good catches). ^a ^Good Catches: hard limit attempts + soft limit pullbacks + SSRC pullbacks; ^b^ Based on 99 data pulls; ^c^ Represents the percentage per type of good catch. Abbreviations: DERS, dose error-reduction software; SD, standard deviation; Min, minimum; Max, maximum; SSRC, single-step rate change

Good Catches ^a,b^ Per 100 DERS Infusions		
Mean	SD	
3.3	1.28	
Median	Min	Max
3.2	1.4	10.6
Average DERS Infusions to One Good Catch Daily	34.5	
Representation of Types of Good Catches Captured (%) ^c^		
Hard Limit Attempt	68.0	
Soft Limit Pullback	18.9	
SSRC Pullback	13.1	

Correlation analyses

Correlation analyses demonstrated weak associations between DL compliance and alert-related outcomes, including overall alert rate, alert subtype rates, override rates, and good catch metrics (Table 5). Statistically significant correlations were observed between DL compliance and the overall alert rate (p = 0.016) as well as the rate of SSRC alerts (p = 0.011); however, these correlations were weak in magnitude (Spearman ρ = -0.242 and -0.256). These findings suggest that higher DL compliance was not strongly associated with reductions in alert burden, override frequency, or rates of reprogramming.

**Table 5:** Correlation with drug library compliance. ^a^ Test statistics are reported as t-values. A two-sided p-value <0.05 was considered statistically significant. ^b ^Calculated using Spearman rank-order correlation. Abbreviations: SSRC, single-step rate change; DERS, dose error-reduction software

	Correlation Coefficient (ρ)	t-value^a^	p-value ^b^
Overall Alert Rate	-0.242	-2.46	0.016
Rate of Hard Limit Attempts	-0.024	-0.239	0.812
Rate of Soft Limit Attempts Alerts	-0.109	-1.08	0.281
Rate of SSRC Alerts	-0.256	-2.61	0.011
Overall Override Rate	-0.141	-1.40	0.164
Soft Limits Override Rate	-0.137	-1.37	0.175
SSRC Override Rate	-0.051	-0.50	0.618
Overall Rate of Alerts Resulting in Reprogramming (Good Catches)	0.135	1.35	0.182
Overall Rate of Good Catches per DERS Infusion	-0.021	-0.211	0.833

A scatter plot analysis of alert rates and override rates for soft limit and SSRC alerts demonstrated considerable variability across institutions (Figure 1). Facilities with similar DL compliance levels showed substantial differences in alert and override rates, suggesting variability in DL configuration and optimization. For example, institutions with comparable DL compliance levels exhibited markedly different alert burdens and override behaviors, indicating potential opportunities for system optimization.

**Figure 1:**
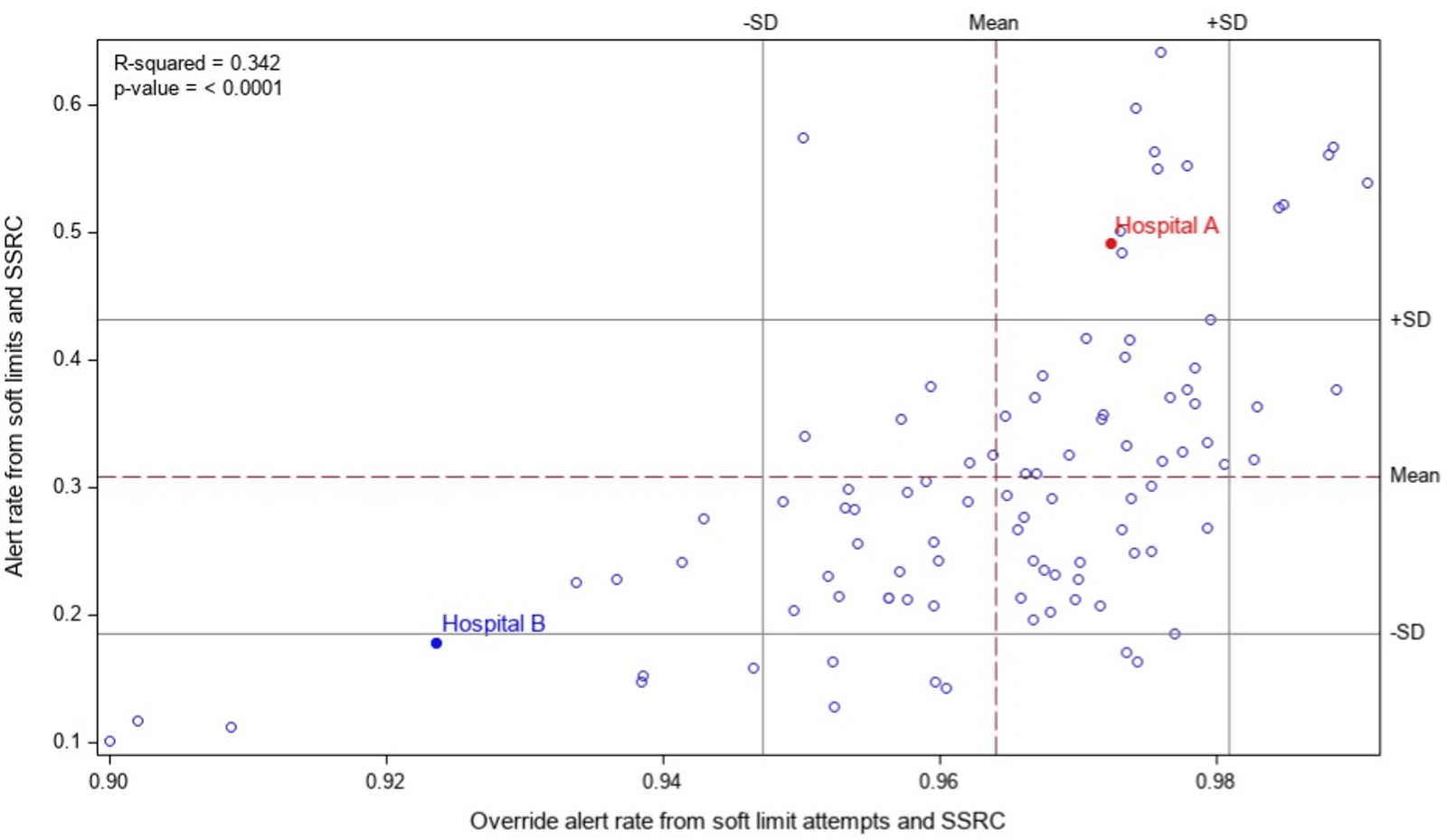
Correlation between alert rate and override rate from soft limit and single-step rate change. Abbreviation: SSRC, single-step rate change; SD, standard deviation

## Discussion

This multicenter retrospective analysis of 13.7 million infusions across 90 institutions evaluated the relationship between DL compliance and alert-related metrics using a benchmarking approach. The overall DL compliance rate of 95.9% exceeded the recommended threshold of 95% [5] and was higher than values reported in prior studies [7]. Despite this high level of compliance, no significant differences in alert rates, override rates, or alerts resulting in reprogramming (“good catches”) were observed between institutions with DL compliance ≥95% and those with lower compliance. In addition, correlation analyses demonstrated only weak associations between DL compliance and alert-related outcomes. Although several correlations were negative, the evidence was insufficient to conclude a statistically significant relationship with DL compliance. Therefore, higher compliance did not appear to be associated with changes in override behavior, hard-limit attempts, or good-catch rates in this dataset. It is important to note that, because this was a retrospective observational benchmarking study, the findings should be interpreted as describing associations between metrics rather than implying causal effects of DL compliance on alert-related outcomes.

The above findings suggest that DL compliance, while an important foundational metric, is insufficient as a standalone indicator of smart infusion pump optimization. Although maintaining high compliance remains a key objective in CQI programs, our results indicate that increasing compliance alone does not necessarily reduce alert burden or improve alert effectiveness. Therefore, additional metrics, including alert frequency, override behavior, and reprogramming events, should be incorporated into routine CQI assessments.

The overall alert rate in this study was high, with approximately one-third of infusions generating alerts and more than 90% of alerts being overridden. These findings are consistent with prior literature reporting high override rates, particularly for soft limit alerts [4,7,10]. High override rates are often indicative of non-actionable or clinically expected alerts, such as those related to appropriate dose titration. Previous studies have demonstrated that optimization of DL limits can reduce clinically insignificant alerts and improve the effectiveness of DERS [4,6]. Together, these findings emphasize the importance of ongoing review and refinement of alert thresholds, including soft limits and SSRC parameters, to minimize unnecessary alerts while maintaining patient safety.

Importantly, alerts resulting in reprogramming (“good catches”) were observed in approximately 3% of DERS infusions, corresponding to one good catch for every 34.5 infusions. This finding suggests that a subset of alerts influences infusion programming behavior and may represent opportunities for error prevention. However, the aggregate data do not permit determination of whether the original programming would have resulted in a clinically significant medication error or patient harm. While hard limit alerts accounted for the majority of reprogramming events, SSRC alerts also contributed to a meaningful proportion of good catches, underscoring their potential role as an additional safety mechanism in infusion pump systems. These findings support the need to preserve clinically relevant alerts while reducing non-actionable alert burden.

Benchmarking across institutions revealed substantial variability in alert rates and override behavior, even among facilities with similar DL compliance levels. This variability suggests that differences in DL configuration, alert threshold settings, and local clinical practices may significantly influence alert burden and system performance. In practical terms, institutions with comparable compliance rates may experience markedly different alert volumes and override patterns, indicating opportunities for optimization through data-driven benchmarking. Comparing performance across institutions may therefore help identify achievable targets for reducing alert burden and improving workflow efficiency.

These findings have important implications for CQI programs. Smart infusion pump data provide a valuable source of real-world evidence for evaluating system performance; however, prior work has shown that such data are not consistently analyzed or acted upon in clinical practice [11]. Integrating benchmarking and routine analysis of alert-related metrics into CQI workflows may support more effective DL optimization and contribute to reductions in alert fatigue. Given that alert fatigue and suboptimal alert configuration are recognized contributors to infusion-related errors [12], leveraging these data may improve both workflow efficiency and patient safety. 

Limitations

This study has several limitations. First, the retrospective analysis included de-identified aggregated CQI data, which limited the ability to evaluate institution characteristics, patient-level or clinical context, including the specific medications associated with alerts and the clinical appropriateness of overrides or reprogramming events. Second, some institutions contributed multiple data extracts representing different observation periods. Although these extracts were analyzed as separate observations, they may not have been fully independent. The statistical analyses did not explicitly account for clustering at the institutional level; therefore, inferential statistical results should be interpreted as exploratory. Nonetheless, because only nine additional extracts were contributed beyond the 90 unique institutions included in the analysis, we do not believe this limitation materially affects the overall descriptive benchmarking findings. Third, the dataset was restricted to a single infusion pump platform, which may limit generalizability to other systems. 

Additionally, the data did not provide insight into clinician decision-making, including reasons for alert overrides or subsequent clinical actions following alerts. Finally, it was not possible to assess whether institutions used CQI findings to implement changes to their DLs or whether such changes resulted in measurable improvements over time.

## Conclusions

Comprehensive analysis of smart infusion pump alert data, including alert rates, override patterns, and alert-associated reprogramming events, may help institutions identify opportunities to optimize DL configurations and evaluate alert burden. While DL compliance remains an important metric, it should be interpreted alongside additional CQI measures when assessing smart infusion pump performance.

These findings highlight the need for broader CQI strategies that incorporate detailed evaluation of alert behavior and DL configuration. Benchmarking across institutions may provide actionable insights to guide optimization efforts, supporting incremental reductions in alert fatigue and more efficient infusion workflows.

## References

[1] Schneider PJ, Pedersen CA, Ganio MC, Scheckelhoff DJ (2019). ASHP national survey of pharmacy practice in hospital settings: Workforce-2018. Am J Health Syst Pharm.

[2] Alamer F, Alanazi AT (2023). The impact of smart pump technology in the healthcare system: a scope review. Cureus.

[3] (2026). Sentinel Event Alert 63: optimizing smart infusion pump safety with DERS. https://www.jointcommission.org/en-us/knowledge-library/newsletters/sentinel-event-alert/issue-63.

[4] Lehr J, Vitoux RR, Evanovich Zavotsky K, Pontieri-Lewis V, Colineri L (2019). Achieving outcomes with innovative smart pump technology: partnership, planning, and quality improvement. J Nurs Care Qual.

[5] (2026). Institute for Safe Medication Practices (ISMP): guidelines for optimizing safe implementation and use of smart infusion pumps. https://www.ismp.org/guidelines/safe-implementation-and-use-smart-pumps.

[6] Mansfield J, Jarrett S (2015). Optimizing smart pump technology by increasing critical safety alerts and reducing clinically insignificant alerts. Hosp Pharm.

[7] Melton KR, Timmons K, Walsh KE, Meinzen-Derr JK, Kirkendall E (2019). Smart pumps improve medication safety but increase alert burden in neonatal care. BMC Med Inform Decis Mak.

[8] Cvach M (2012). Monitor alarm fatigue: an integrative review. Biomed Instrum Technol.

[9] Korniewicz DM, Clark T, David Y (2008). A national online survey on the effectiveness of clinical alarms. Am J Crit Care.

[10] Marwitz KK, Giuliano KK, Su WT, Degnan D, Zink RJ, DeLaurentis P (2019). High-alert medication administration and intravenous smart pumps: a descriptive analysis of clinical practice. Res Social Adm Pharm.

[11] (2026). Institute for Safe Medication Practices (ISMP): survey results: smart pump data analytics-pump metrics that should be monitored to improve safety. https://www.ismp.org/sites/default/files/attachments/2018-07/20180712.pdf.

[12] Carlson R, Johnson B, Ensign RH 2nd (2015). Development of an "infusion pump safety score". Am J Health Syst Pharm.

